# Functional and Structural Characterization of a Potent C1q Inhibitor Targeting the Classical Pathway of the Complement System

**DOI:** 10.3389/fimmu.2020.01504

**Published:** 2020-07-17

**Authors:** Nick S. Laursen, Dennis V. Pedersen, Heidi Gytz, Alessandra Zarantonello, Jens Magnus Bernth Jensen, Annette G. Hansen, Steffen Thiel, Gregers R. Andersen

**Affiliations:** ^1^Department of Molecular Biology and Genetics, Center for Structural Biology, Aarhus University, Aarhus, Denmark; ^2^Department of Clinical Immunology, Aarhus University Hospital, Aarhus, Denmark; ^3^Department of Biomedicine, Aarhus University, Aarhus, Denmark

**Keywords:** complement system, nanobody, antibody, inhibitor, C1q, crystal structure

## Abstract

The classical pathway of complement is important for protection against pathogens and in maintaining tissue homeostasis, but excessive or aberrant activation is directly linked to numerous pathologies. We describe the development and *in vitro* characterization of C1qNb75, a single domain antibody (nanobody) specific for C1q, the pattern recognition molecule of the classical pathway. C1qNb75 binds to the globular head modules of human C1q with sub-nanomolar affinity and impedes classical pathway mediated hemolysis by IgG and IgM. Crystal structure analysis revealed that C1qNb75 recognizes an epitope primarily located in the C1q B-chain that overlaps with the binding sites of IgG and IgM. Thus, C1qNb75 competitively prevents C1q from binding to IgG and IgM causing blockade of complement activation by the classical pathway. Overall, C1qNb75 represents a high-affinity nanobody-based inhibitor of IgG- and IgM-mediated activation of the classical pathway and may serve as a valuable reagent in mechanistic and functional studies of complement, and as an efficient inhibitor of complement under conditions of excessive CP activation.

## Introduction

The complement system plays an important role in maintaining homeostasis by removal of apoptotic cells and pathogens (1). The classical pathway (CP) of complement is activated by binding of the C1 complex to a number of ligands, including immunoglobulin G (IgG) and immunoglobulin M (IgM) bound to surface antigens or in immune complexes. The C1 complex consists of the pattern recognition molecule C1q associated with a tetramer of the proteases C1r and C1s. C1q is a multimeric molecule with six heterotrimeric collagen helices made up by the A, B, and C-chains (2). The N-terminal collagen regions in the three C1q subunits form heterotrimers that hexamerize to form intact C1q, and these collagen regions also firmly hold C1r and C1s attached. The collagen helices are tightly packed at the N-terminus but diverge toward the C-terminus into six individual collagen stems that end in trimeric C-terminal globular heads (gC1q). Each chain of gC1q adopts a ten-stranded β-sandwich fold that together form the highly compact spherical structure of gC1q (2–4).

The gC1q mediates the C1q interaction with numerous ligands including the Fc-region of antigen-bound IgG and IgM (5–7). Recognition by C1q leads to autoactivation of C1r which cleaves and activates C1s. Activated C1s cleaves C4 into C4b, that upon binding of zymogen C2 forms the C4bC2 proconvertase complex. This proconvertase is also cleaved by C1s resulting in appearance of the CP C3 convertase C4bC2a (1). The CP C3 convertase cleaves C3 into C3a and C3b. C3b is deposited at the activator surface and drives the amplification of the alternative pathway (AP) of complement resulting in opsonization and phagocytosis. In contrast, the soluble C3a acts as a modulator of inflammation targeting a range of immune as well as non-immune cells. At sufficiently high C3b densities on cells, activation of the terminal pathway (TP) may lead to assembly of the membrane attack complex (MAC) and complement dependent cytotoxicity (CDC) (1).

The best characterized ligands for C1q are immune complexes containing IgG and IgM. The affinity between C1q and a single IgG molecule is low (7). However, on a surface, antigen-bound IgGs may oligomerize into large ordered hexameric structures that enable multivalent binding of C1q and C1 activation (8). The recognition of IgG involves residues in the B- and C-chains of gC1q, that interact with both CH2 domains of IgG (9–15). In contrast to IgG, IgM circulates primarily as pentamers and a smaller amount of hexamers. Mutational studies and cryo-electron tomography structures of IgM in complex with C1 suggest that the binding site of C1q is located in the IgM Cμ3 domain (16, 17) with residues in the B-chain of gC1q being involved in the interaction (18, 19).

Initiation of the CP can lead to opsonization, cell lysis and may induce a potent inflammatory response, and as a consequence, undesirable CP activation can lead to pathology. CP activation can result in allograft rejection following transplantation (20) but can also drive a number of autoantibody mediated diseases including neuromyelitis optica (NMO) (21, 22), generalized myasthenia gravis (gMG) (23, 24), and cold agglutinin disease (CAD) (25). Autoantibodies can bind to self-surfaces or be deposited as immune complexes and activate the CP resulting in tissue damage and inflammation. In NMO and gMG, IgG autoantibodies against aquaporin-4 (22) and acetylcholine receptors, respectively, activate the CP which leads to CDC mediated injury to astrocytes in the case of NMO (26) or at the neuromuscular junction in gMG (24). CAD is usually caused by IgM autoantibodies against the “I” antigen on erythrocytes (25) and results in C3 deposition on erythrocytes and extravascular hemolysis by macrophages of the reticuloendothelial system in the liver (27, 28). Inhibition of CP with an anti-C1s mAb (Sutimlimab) abrogated extravascular hemolysis in CAD patients supporting an important role of complement as a pathogenic factor in CAD and the CP as a therapeutic target (29).

With the realization that complement is involved in numerous disease states, therapeutic strategies to modulate specific complement components are gaining increased attention (30). Despite the recognized contributions from CP and the related lectin pathway (LP) to pathogenesis, the AP and TP have primarily been targeted. Here, we present a C1q specific nanobody (C1qNb75) that inhibits CP activation driven by IgG and IgM. Nanobodies are single domain antibodies derived from the variable domain of camelid heavy-chain only antibodies. We demonstrate that C1qNb75 binds with sub-nanomolar affinity to human gC1q. It directly blocks C1q's interaction with IgG, and prevents activation of complement via IgG and IgM ligands, and antibody-mediated hemolysis.

## Results

### Nanobody Generation and Characterization

**Figure 1:**
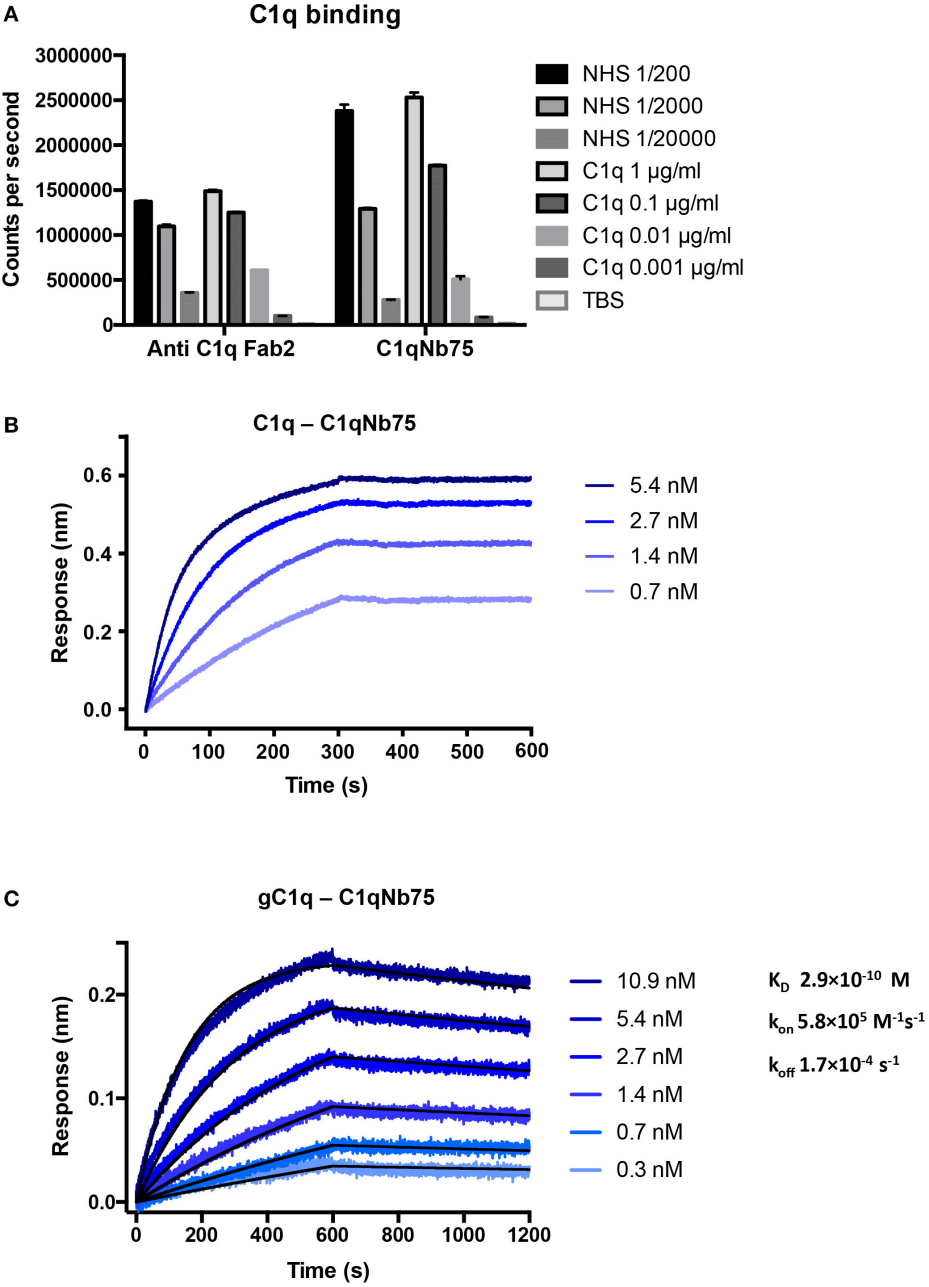
C1qNb75 binds with high affinity to human C1q. **(A)** Solid-phase immunoassay (TRIFMA) with anti-C1q Fab2 or C1qNb75 coated in microtiter wells. The binding of purified C1q or C1q in NHS is measured. The signal is given as counts per second after measurement by time-resolved fluorometry. Shown are mean and SD. **(B)** Bio-layer interferometry (BLI) measurement of the interaction between purified C1q in solution and immobilized C1qNb75. **(C)** As in previous panel but showing the interaction between recombinant gC1q and immobilized C1qNb75 with calculated k_on_, k_off_, and K_D_. The Experimental data (blue curves) was globally fitted to a 1:1 binding model (black curves).

To isolate nanobodies (Nbs) against C1q we immunized a llama with a mixture of human C1q and gC1q. Peripheral blood mononuclear cells were isolated and a phage display library presenting the Nbs as fusion proteins fused to the PIII phage coat protein, was generated. After two rounds of *in vitro* selection, we obtained a number of clones, which were subsequently cloned into a bacterial expression vector, expressed and purified. After initial characterization of the nanobodies's ability to inhibit IgG mediated complement activation (Figure S1A) we identified C1qNb75 as a potent CP inhibitor and we decided to further characterize this Nb. Binding of recombinant C1qNb75 to C1q was first evaluated by a sandwich-type solid-phase immunoassay using C1qNb75 coated in microtiter wells at different concentrations and purified C1q or normal human serum (NHS) as a source of C1q (Figure 1A). Immobilized C1qNb75 captured both purified C1q and C1q from NHS in a dose-dependent manner. The interaction was further characterized using bio-layer interferometry (BLI). We coupled C1qNb75 to amine reactive sensors and the interaction was assayed using C1q purified from human plasma (Figure 1B). Increasing signal was observed for increasing concentrations of C1q. As C1q is a homo-hexamer, and one molecule of C1q may bind up to six Nb molecules simultaneously, we did not attempt to calculate the binding constants. Instead, we used BLI to evaluate binding of immobilized C1qNb75 to monomeric single-chain recombinant gC1q (Figure 1C). The Nb bound gC1q with very high affinity (K_D_ = 0.3 nM) and an off-rate of 1.7 × 10^−4^ s^−1^ corresponding to a t_1/2_ = 4.1 × 10^3^ s for a first-order dissociation of the monomeric interaction. Comparison of the dissociation phases for C1q and gC1q confirms that C1q binds with avidity to immobilized C1qNb75 since the C1q dissociation is characterized by an extremely slow dissociation phase as compared to the monovalent interaction (Figures 1B,C).

### C1qNb75 Blocks Binding of C1q to IgG

**Figure 2:**
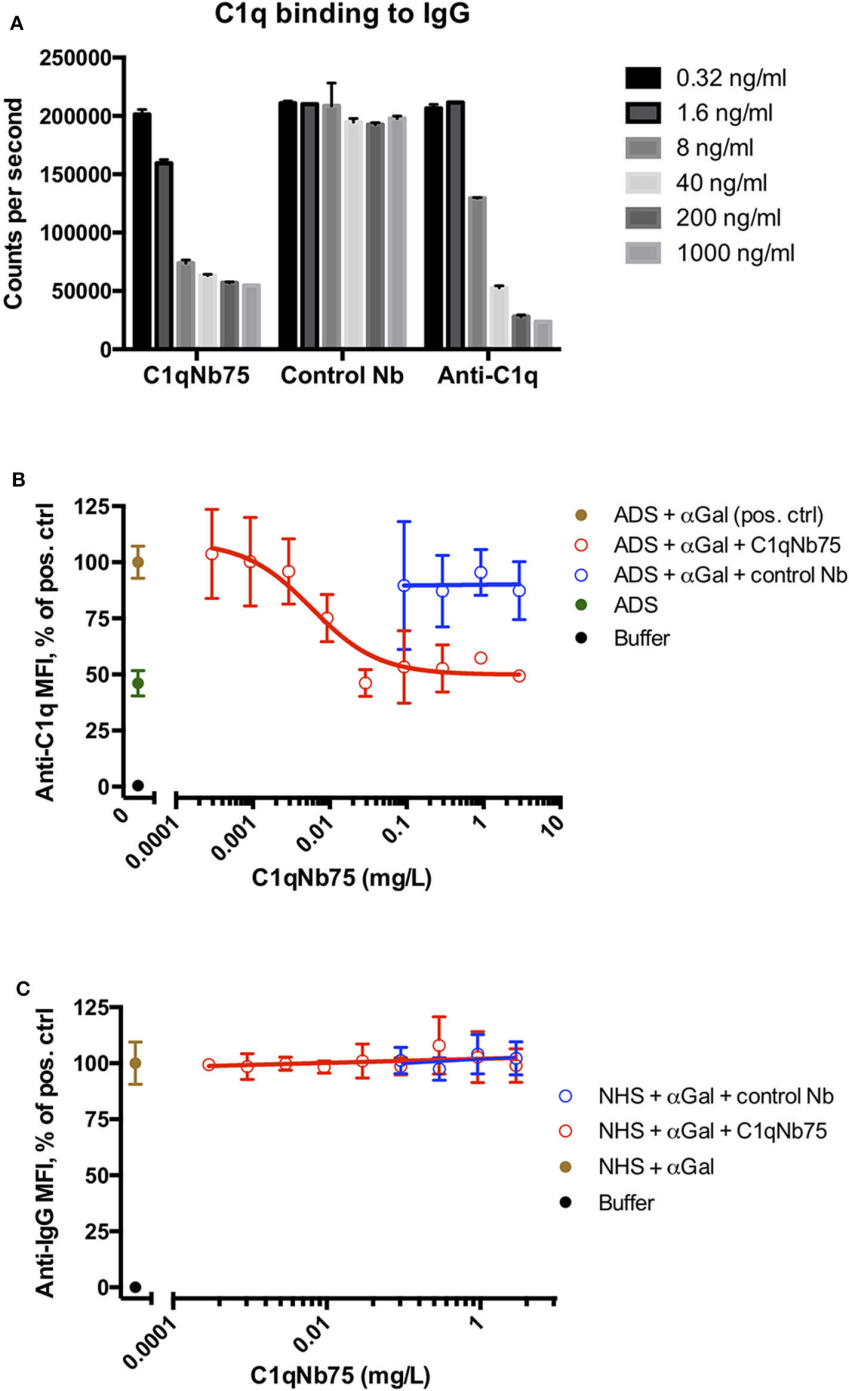
C1qNb75 interferes with binding of C1q to surface bound IgG. **(A)** Binding of C1q to IgG in microtiter wells in the presence of the indicated contractions of C1qNb75, control Nb or anti-C1q antibody. **(B)** Binding of C1q (in ADS) to antibody sensitized pig red blood cells in the presence of C1qNb75 or control Nb. C1q binding was detected by flow cytometry using a polyclonal fluorescently labeled anti-C1q antibody and plotted as % of mean fluorescence intensity (MFI) of a positive control with SD. Results are representative of two independent experiments. **(C)** Same experiments as in panel **(B)**, but where binding of IgG was measured with flow cytometry using fluorescently labeled anti-IgG.

The CP can be activated by binding of C1q to immune complexes containing complement activating antibodies. We assayed the ability of C1q in NHS to bind to surface immobilized human IgG in the presence of increasing concentrations of C1qNb75 with a solid-phase immunoassay (Figure 2A). Human IgG was immobilized in microtiter wells and incubated with 0.2% NHS in the presence of variable concentrations of C1qNb75, control Nb or IgG anti-C1q. This was followed by detection of bound C1q with biotin labeled anti-C1q antibody. C1qNb75 clearly inhibited binding of C1q in a concentration dependent manner like the anti-C1q IgG, whereas no inhibition was observed with the control Nb.

We next addressed if C1qNb75 inhibited C1q docking to IgG antibodies bound on antigens. To do this, we used human antibody deficient serum (ADS) as source of C1q, affinity purified human IgG antibodies recognizing terminal galactose-α-1,3-galactose (IgG anti-αGal) and pig red blood cells (RBCs), which express the relevant carbohydrate structure. These RBCs present ~6 × 10^4^ terminal galactose-α-1,3-galactose residues per cell (31) and efficiently bind the affinity purified antibody preparation (32). Using flow cytometry, we found that increasing concentrations of C1qNb75 caused decreasing C1q deposition on pig RBCs (Figure 2B), supporting blockage of C1q docking on antigen-bound antibodies by the Nb. In accordance with the K_D_ value observed by BLI, the inhibition curve had an inflection point below a C1qNb75 concentration of 0.01 mg/L, corresponding to a nanobody concentration of 0.7 nM. A control Nb of irrelevant specificity at similar concentrations did not displace C1q from the RBCs (Figure 2B). Moreover, neither C1qNb75 nor the control Nb influenced antibody binding on pig RBCs in NHS (Figure 2C), in accordance with selective blockade of C1q's ability to dock on immunoglobulins. Interestingly, even at high Nb concentrations, C1q binding was not inhibited to a level comparable to that of buffer alone, but plateaued at a level similar to that of ADS alone (Figure 2B). This suggests that C1q may bind some ligand on pig RBCs even in the presence of C1qNb75. Whether C1q binds pig RBCs through a site in the gC1q domain not concealed by C1qNb75 or via its collagen tail needs further investigation. Overall these experiments demonstrated that C1qNb75 blocks C1q docking on aggregated IgG and IgG bound to antigens.

### Inhibition of Antibody Mediated CP Activation and Hemolysis

**Figure 3:**
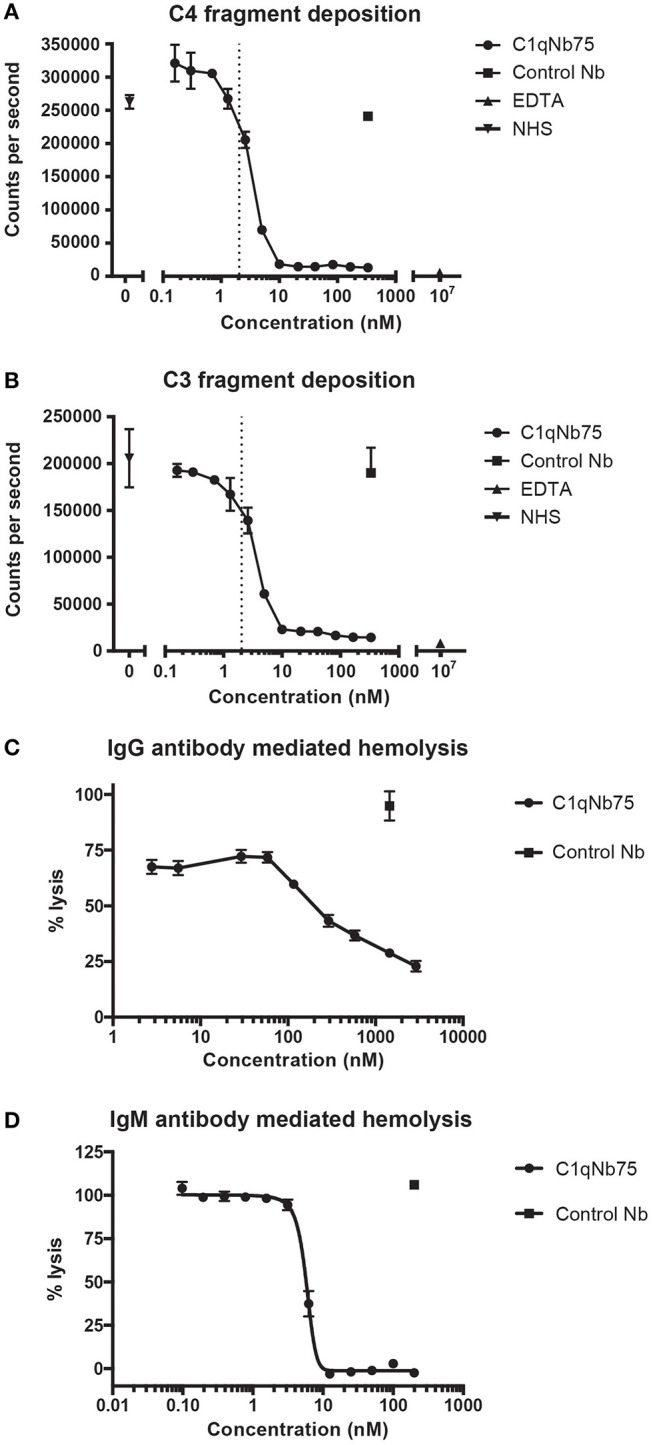
C1qNb75 inhibits IgG and IgM mediated activation of the classical pathway and hemolysis. **(A)** Deposition of C4 fragments on IgG coated surface from 0.2% NHS in the presence of increasing concentrations of C1qNb75, control Nb (333 nM) or EDTA (10 μM). Shown are mean and SD of two independent experiments. The dotted line indicates the calculated Nb concentration resulting in a 1:1 binding to gC1q. **(B)** As in panel A, but measuring C3 fragment deposition. **(C)** Hemolysis of sheep RBCs sensitized with IgG in the presence of increasing concentrations of C1qNb75, control Nb or EDTA. Data are plotted as % lysis where 100% lysis is incubation of RBCs with water. Results are representative of two similar experiments. Shown are mean and SD. **(D)** Hemolysis of sheep RBCs sensitized with IgM in the presence of increasing concentrations of C1qNb75, control Nb or EDTA. Data are plotted as % lysis where 100% lysis is incubation of RBCs with water. Results are representative of three independent experiments. Shown are mean and SD.

The classical pathway is activated by both IgG and IgM, and we first evaluated the effect of C1qNb75 on CP activation by IgG. CP activation was assayed by measuring C4 and C3 fragment deposition in IgG coated microtiter wells with NHS as a source of complement. C1qNb75 potently inhibited C4 deposition in a concentration-dependent manner (Figure 3A). Consistent with C3 being downstream of C4 in the CP, C1qNb75 also inhibited C3 deposition (Figure 3B). No C4 and C3 deposition occurred when divalent metal ions were chelated from the NHS with 10 mM EDTA. The presence of control Nb (333 nM) had no effect on C4 and C3 deposition compared to incubation with NHS only (Figures 3A,B). A sharp drop in C3 and C4 deposition was observed at a C1qNb75 concentration of 2–4 nM, roughly corresponding to six times the calculated concentration of C1q in the assay assuming a serum concentration of C1q at 170 nM (2.04 nM, dotted line in Figures 3A,B). If the K_D_ = 0.29 nM observed by BLI is applicable in this serum-based experiment, and binding of the nanobody to its six binding sites on C1q is non-cooperative, it appears that C1qNb75 must bind at least four gC1q's to efficiently inhibit IgG mediated CP activation.

Depending on the level of complement regulation, activation of CP may result in TP activation and MAC formation. As an indirect assessment of MAC formation in the presence of C1qNb75, we performed hemolysis assays with IgG antibody sensitized sheep RBCs and NHS as source of complement. C1qNb75 blocked IgG mediated hemolysis in a concentration-dependent manner while no effect was observed when incubating with a control Nb (Figure 3C). We next asked whether C1qNb75 could also inhibit hemolysis due to CP activation driven by IgM. Though IgG and IgM share homology and are both ligands for C1q, C1q recognizes IgG and IgM differently (9, 17, 19). Thus, C1qNb75 could potentially block C1q binding to IgG without affecting binding to IgM. To investigate the influence of the nanobody on IgM mediated CP activation, IgM-sensitized sheep RBCs were incubated with NHS in the presence of C1qNb75 or control Nb and hemolysis was measured (Figure 3D). C1qNb75 strongly inhibited IgM mediated hemolysis in a concentration-dependent manner with a half maximal inhibitory concentration (IC50) of 5.8 nM. Addition of control Nb of irrelevant specificity did not inhibit hemolysis (Figure 3D). To investigate if C1qNb75 binding to C1q itself can activate complement, we measured C5a generation in NHS after addition of the Nb. As shown in Figure S1B, increasing C1qNb75 concentrations had no effect on C5a generation. Collectively the data show that C1qNb75 inhibits IgG and IgM mediated complement activation and CDC as assayed by sheep RBC lysis.

### C1qNb75 Sterically Prevents the Interaction Between C1q and Antibodies

**Figure 4:**
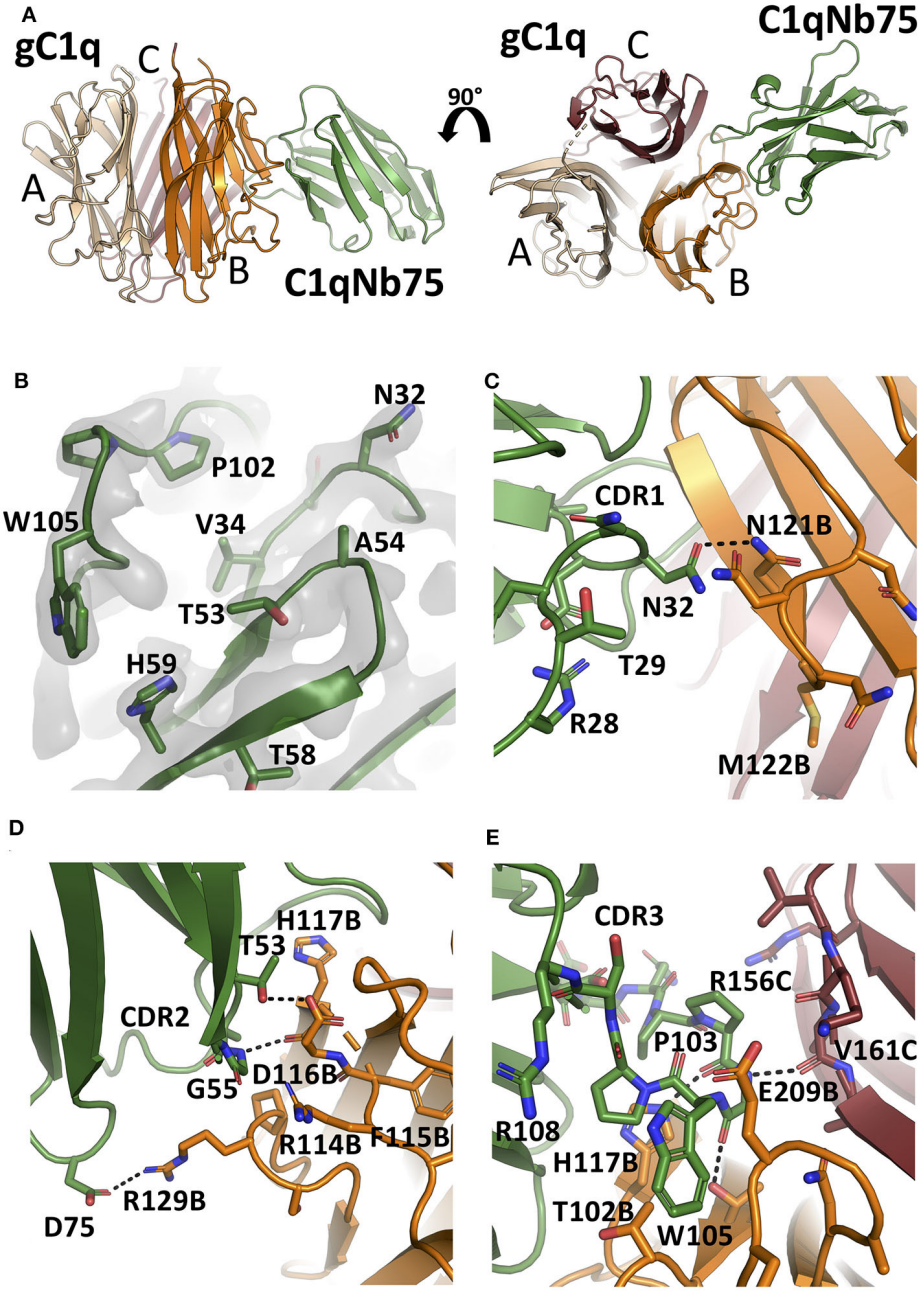
The crystal structure of the gC1q-C1qNb75 complex reveals the epitope. **(A)** Side view (left) and top view (right) of the gC1q-C1qNb75 complex. The different subunits of gC1q are colored in wheat (A-chain), orange (B-chain) and brown (C-chain) whereas C1qNb75 is displayed in green. **(B)** Omit 2mF_o_-DF_c_ electron density map contoured at 1.2σ around the three CDRs. Residues shown with side chains in sticks were omitted for map calculation. **(C–E)** The interface between gC1q and C1qNb75 with selected residues shown in sticks. **(C)** CDR1 contacts, **(D)** CDR2 contacts, **(E)** CDR3 contacts. Hydrogen bonds are shown with black dashes. The gC1q residues are labeled with their chain identifier as suffix.

To unravel the molecular details of the interaction between C1qNb75 and gC1q we determined the crystal structure of C1qNb75 in complex with gC1q at 2.2 Å resolution (Figure 4A and Table S1). Clear electron density was observed for the vast majority of residues in gC1q and C1qNb75 (Figure 4B) which allowed us to model the intermolecular interface with high confidence. PISA analysis (33) revealed an epitope with an area of 740 Å^2^ on C1q, which is in the low end for such complexes, whereas the observed shape complementarity of 0.74 is in the average range (34). In the crystal structure, there are two complexes in the asymmetric unit with the two gC1q copies being almost identical. Minor differences observed in two loops of the C1q A-chain are likely caused by crystal packing, and these loops are located far from the C1qNb75 epitope. Superposition of the complex with the structure of single-chain gC1q (PDB ID: 5HKJ) reveals no major conformational changes in C1q upon Nb binding (Figure S1C) suggesting that conformational changes in gC1q induced upon nanobody binding do not contribute to the inhibitory mechanism of C1qNb75.

**Figure 5:**
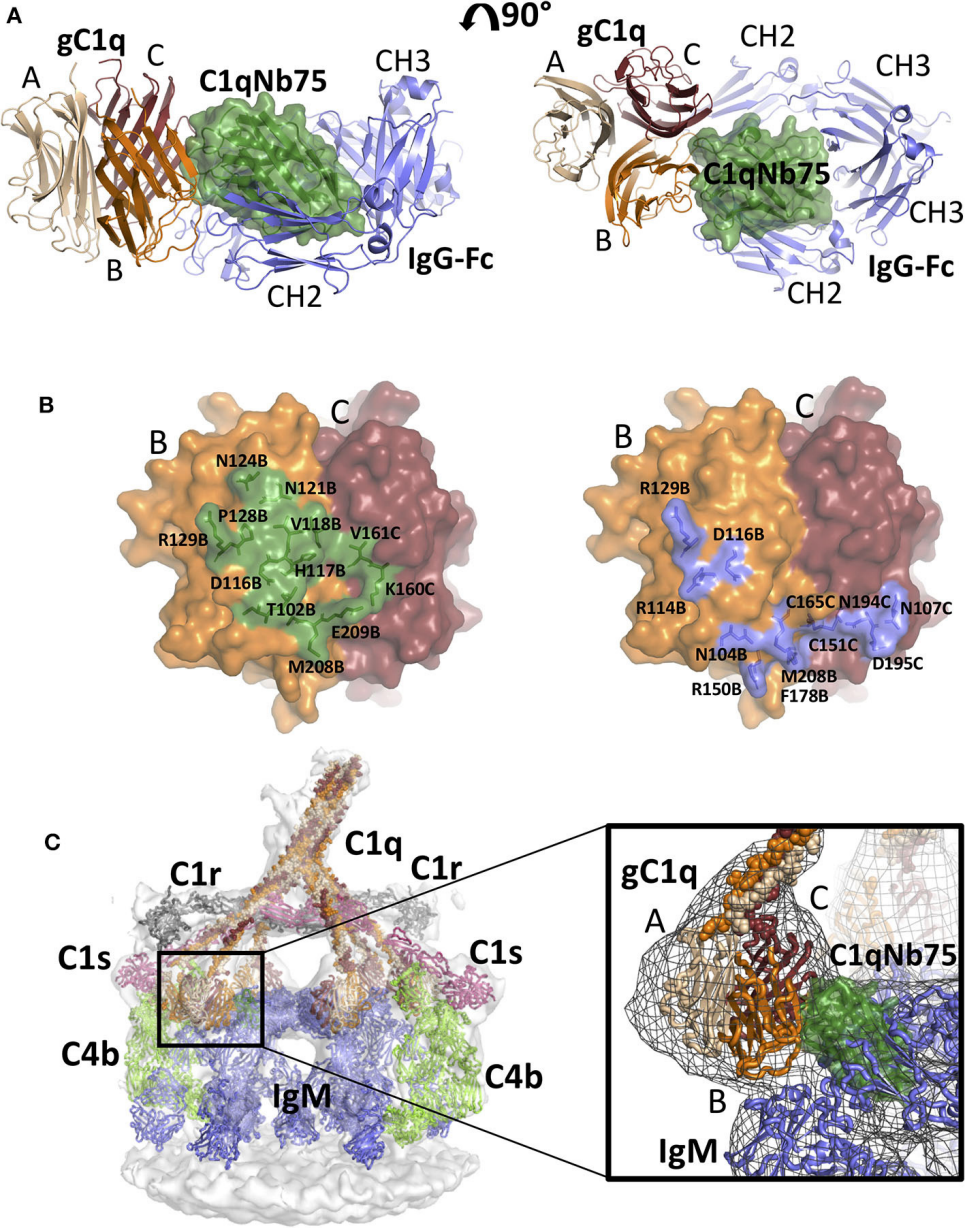
Comparison of the gC1q-C1qNb75 complex with models of IgG Fc-gC1q and C1-IgM-C4b explains the inhibition of the CP. **(A)** Alignment of the crystal structure of the gC1q-C1qNb75 complex with the cryo-EM structure of IgG Fc in complex with gC1q (PDBID: 6FCZ). The gC1q-C1qNb75 is colored as in Figure 4 and IgG-Fc is shown in blue. Similar to Figure 4A, side view (left) and top view (right) are shown. A massive clash between C1qNb75 and IgG Fc is observed. **(B)** The C1qNb75 epitope overlaps with gC1q residues involved in IgG contacts. Left: epitope of C1qNb75 on gC1q with residues involved in the interaction shown as sticks and in green. Right: gC1q residues involved in the interaction with IgG-Fc are shown in sticks and colored blue. **(C)** Model of the C1-IgM-C4b complex and comparison with the structure of gC1q-C1qNb75. Left: Overview of the entire C1-IgM-C4b complex, right: close up view of the steric overlap of C1qNb75 with IgM Fc. The C4b is colored light green, C1r gray, C1s purple, IgM blue, whereas C1qNb75 and C1q chains are colored as in **(A)**. The 3D reconstruction is displayed as a transparent surface in the overview (left) and as a mesh in the close-up (right).

C1qNb75 binds to the side of gC1q and is primarily contacting residues in the B-chain of C1q supplemented with few contacts to the C-chain. The paratope is made up of residues from all three CDR regions of C1qNb75. In CDR1, Asn32 forms hydrogen bonds with Asn121B (letter after residue number indicates C1q chain, mature numbering of C1q is used) (Figure 4C). CDR2 residues Thr53 and Gly55 form hydrogen bonds with C1q residue Glu116B next to Arg114B, which are both involved in the interaction between C1q and IgG (9) (Figure 4D). Asp75 in the Nb framework region 3 forms a salt bridge with C1q Arg129B, which is also involved in the interaction with IgG (9) (Figures 4D, 5B). A hydrophobic patch on CDR3 formed by residues Pro102, Pro103, Trp105, Pro106 protrudes from the β-sandwich immunoglobulin fold and is inserted in a C1q cavity delimited by the C1q-B chain loop carrying Glu209B, and the loop carrying Thr102B (Figure 4E). The latter residue is close in sequence to Arg108B and Arg109B, known to mediate interactions with pentraxin 3 and IgM (18). The cavity is likely shielded from the solvent due to proximity of C1qNb75 Arg108 to Glu209B on one side, and the presence of gC1q Arg156C on the other side (Figure 4E). Hydrogen bonds are formed between Pro103 and His117B, and from Gly104 to Val161C and Thr100B. In addition, there are multiple water molecules bridging C1qNb75 with gC1q. Sequence alignment with mouse C1q B-chain explains the absence of mouse cross reactivity (Figure S2A). The most prominent amino acid difference is at position 117, where the histidine is replaced by a lysine in mouse C1q B, introducing a positively charged and much larger side chain, which likely disrupts the hydrophobic pocket housing Trp105 in human C1q (Figure S2B).

As demonstrated in Figures 2, 3, C1qNb75 potently inhibits C1q binding to IgG and IgM. gC1q binds to the CH2 domains of IgG near the Fab-Fc hinge (10, 11, 15) using residues located in the B- and C-chains of C1q (9, 12–14). To analyze how C1qNb75 prevents C1q binding to IgG, we superimposed our structure on that of IgG-Fc bound to gC1q (9) (Figure 5A). This structural comparison reveals that C1qNb75 blocks binding of C1q to IgG by steric hindrance, restraining access to critical C1q B-chain residues important for recognition of one CH2 domain of IgG Fc. (Figure 5B). In contrast, C1qNb75 does not directly block the area on C1q involved in binding to the second CH2 domain of IgG Fc.

With respect to IgM binding, C1q is suggested to interact with the IgM-Fc Cμ3 domains using residues Arg108B, Arg109B, Arg150B, and Phe178B (17). To examine how C1qNb75 prevents C1q binding to IgM we first generated an atomic model for C1 bound to IgM and C4b by fitting known structures into a deposited 3D cryo-electron tomography reconstruction (EMDB-4878 and Table S2) (17), and subsequently aligned the C1qNb75-gC1q complex structure to this model. In this model, C1qNb75 exerts extensive steric clashes with the IgM Cμ3 domains (Figure 5C). Collectively, the C1qNb75-gC1q complex structure and the structural comparisons demonstrate that C1qNb75 binds an epitope on C1q directly involved in recognition of both IgG and IgM and explains how C1qNb75 inhibits complement activation.

## Discussion

C1q is the pattern recognition molecule of the C1 complex that initiates the CP of the complement cascade. Recognition of a plethora of molecular patterns by C1q induces C1r and C1s activation resulting in C4 cleavage and assembly of the CP C3 convertase (35–37). Structural and functional studies have provided information on how C1q recognizes the Fc-fragments of IgG and IgM (9, 10, 14, 16–19, 38). gC1q binds a distinct site in each of the two CH2 domains of IgG1. One binding site consists of the area around loop FG of CH2 that interacts with C1q-B chain residue Phe178B and C1q-C residue Asp195C. The other site is formed by loop BC and loop DE of the second CH2 domain proposed to interact with C1q-B residues Arg114B and Arg129B. Our structural data shows that C1qNb75 binds to an epitope on gC1q that overlaps with the binding site of IgG Fc CH2 and thus blocks docking of C1q onto IgG. Likewise, our model suggest that C1qNb75 prevents binding of C1q to antigen-bound IgM by steric hindrance restraining access to residues on gC1q which are involved in the interaction between gC1q and IgM Cμ3.

Our functional data show that C1qNb75 is a potent inhibitor of antibody-mediated CP activation. Thus, C1qNb75 could represent an initial candidate molecule for further development of a therapeutic agent for treatment of autoantibody-mediated conditions, like CAD, NMO, and gMG, where complement contributes to disease pathogenesis. Targeting complement at the site of C1 activation would inhibit MAC-induced membrane damage, but also C3b deposition and associated effects. In the case of CAD this includes C3b mediated extravascular hemolysis. However, complement also plays a role in stimulating adaptive immunity. Antigens coated with deposited C3 fragments (e.g., membrane fragments with iC3b) can lower the threshold for B-cell activation via complement receptor 2 (CR2) and also increase the time antigens are presented by CR2-expressing follicular dendritic cells to B-cells (39–41). Thus, while the immediate injury in gMG and NMO is caused by CDC, complement, through iC3b, may also indirectly augment the generation of new autoantibodies and exacerbate the disease on a longer term.

Since the C1q has a role both in pathogen clearance and in tissue homeostasis by facilitating clearance of apoptotic cells through phagocytosis, long term systemic C1q inhibition needs to be carefully evaluated (42). Genetic deficiencies in CP components C1q, C4 and C2 strongly associate with the autoimmune disease systemic lupus erythematosus (SLE) (43), that may be caused by the failure of macrophages to phagocytose apoptotic cells (44). However, no adverse effects were observed by administration of a C1s specific antibody (sutimlimab) for 2.5 years (29) suggesting that longer term blockade of the CP is safe. In this respect, it is also important to consider that there are functions of C1q for which the activity of C1s and CP activation appears not to be required (45) but may be influenced by a C1q specific antibody like C1qNb75 or the IgG4/Fab ANX005/ANX007 (46).

C1qNb75 does not bind to mouse C1q (data not shown). This prevents application of the C1qNb75 in murine animal models of complement associated pathogenesis unless mouse C1q is fully or partially replaced by human C1q as described in (47). Sequence alignment of the gC1q epitope from common model animals (Figure S2A) suggests that C1qNb75 can be useful in non-murine animal models including primate models of delayed graft function (48) and a pig model of ischemia-reperfusion injury (49). Thus, C1qNb75 may be a valuable tool for assessing the contribution of CP-mediated complement activation to diseases driven by allo- and autoantibodies, but also to evaluate complements contribution to the efficacy of therapeutic antibodies.

The biological functions of C1q extend beyond activation of the classical pathway by immunoglobulins and reach the contexts of tumor growth, neurobiology, pregnancy, hemostasis and immune tolerance as reviewed in (5). Although there are patterns like apoE that are reported to bind C1q via other sites than gC1q (50), the globular heads are required for recognition of the majority of C1q binding patterns including C-reactive protein, pentraxins, LPS, apoptotic cells, DNA, amyloid-β fibrils, DC-SIGN, gC1qR, calreticulin, the Frizzled receptors, and the macrophage receptor SIGN-R1 (5, 6, 51–54). While the interactions with IgG and IgM are well characterized, much less is known about the molecular surfaces of gC1q involved in recognition of non-immunoglobulin ligands (55–57). We propose to use C1qNb75 as a molecular tool to help decipher interactions between C1q and its ligands. Due to their small size, competition experiments with Nbs are likely to give rise to less long-range steric hindrance as compared to larger competitors, e.g., a conventional IgG or its Fab fragment. For this reason, Nbs offer a more accurate tool for mapping areas of interaction in such competition experiments. We envision C1qNb75 could be especially useful to study the involvement of C1q in synaptic pruning and in neurodegenerative diseases like Alzheimer's disease (AD) and glaucoma, where the molecular structures recognized by C1q are still largely unknown as discussed below.

C1q as well as other components of the CP are critical in the developing brain, and mice deficient in C1q, C4, C3, or CR3 have defects in synaptic connectivity (58). During development, binding and activation of C1q on less active synapses leads to deposition of C3 degradation products that are recognized by CR3-expressing microglia which phagocytize the opsonized presynaptic terminals (59–61). However, the same pathway that ensures correct development by pruning excess synapses, seems to be involved in neurodegenerative conditions like AD (62). C1q and C3 are upregulated in AD mouse models and deposited complement components have been found in AD patients (63–66). In AD, C1q binds to vulnerable synapses and activates the CP. This leads to C3 deposition on synapses which are phagocytosed by CR3-bearing microglia. C1 inhibition by antibodies or C1q KO prevents synapse loss following injection of oligomeric amyloid-β (62). Similarly, C3 and CR3 KO rescues synapse loss in mice challenged with oligomeric amyloid-β (62). It is not known whether C1q binds directly to amyloid-β in these models or the presence of amyloid-β exposes other patterns on the synapse that are recognized by C1q. Likewise, little is known about the patterns bound by C1q during synaptic pruning. Thus, whether C1qNb75 blocks binding of C1q to the molecular structures exposed during development and/or in neurodegenerative diseases, like AD, remains to be determined.

With respect to *in vitro* applications, C1qNb75 may become a valuable tool for both functional studies and diagnostics. Due to the very high affinity of immobilized C1qNb75 for C1q, the Nb appears suited for capture and quantitation of C1q in biological samples like serum and cerebrospinal fluid. Our experiments also provide evidence for its application in flow cytometry studies to investigate competition with cell bound C1q binders. Finally, C1qNb75 specifically blocks the CP in functional assays and may thus allow investigators to unravel the specific contributions to complement activation from the CP and LP without depending on protein depletion or genetic knockouts.

In conclusion, we present a potent and high affinity inhibitor of the classical pathway of complement, that is based on the small and versatile Nb scaffold, and define its mode of inhibition through a combination of functional assays and structural biology. C1qNb75 stands out as a powerful tool for investigating CP-mediated complement activation in different contexts, which could possibly help determine binding modes on activators for which the interacting regions on gC1q are unknown. We further suggest possible applications of C1qNb75 as a diagnostic tool, and as a molecule that will efficiently inhibit C1q *in vivo* under conditions of excessive antibody-mediated CP activation.

## Materials and Methods

### Purification of Human C1q

C1q was purified from human outdated plasma using a procedure adapted from (67). Three bags of plasma were thawed at 4°C overnight to allow clotting, 5 mM EDTA was added to the plasma and the precipitate was collected at 6,000 g for 30 min. The supernatant was gaze filtered prior to 2 h incubation at room temperature (RT) with 100 mL Bio-Rex70^TM^ beads (Bio-Rad) equilibrated in 50 mM Na2HPO4, 82 mM NaCl, 2 mM EDTA pH 7.4 (buffer A). The beads were washed extensively with buffer A and packed on a XK16 (GE Healthcare) column. The column was washed with buffer A until baseline and eluted with a 300 mL linear gradient from 82 to 300 mM NaCl. The fractions containing C1q were pooled and a 1:1 v/v ratio of 66% saturated (NH_4_)_2_SO_4_ solution was dripped into the sample while stirring at 4°C. After 15 h, the precipitate was pelleted by centrifugation at 3,000 × g for 30 min and purified C1q was resuspended in 50 mM Tris-HCl, 500 mM NaCl pH 7.2 (storage buffer) and dialyzed three times for at least 6 h at 4°C against 2.5 L of storage buffer before flash freezing at −80°C.

### Selection of C1qNb75

Immunization, library generation and selection was performed essentially as described in (34). A llama was immunized four times each with a 100 μg mixture of purified C1q and gC1q, the latter generated by collagenase digestion of C1q (3). After 84 days blood was collected and peripheral blood mononuclear cells were isolated using Ficoll® Paque Plus (GE Healthcare). RNA was purified using a RNeasy® Plus Mini Kit (Qiagen) following the manufacture's protocol. cDNA was generated by reverse transcription with the Superscript®III First-Strand kit (Invitrogen). Genes were amplified by PCR with VHH specific primers and cloned in to a phagemid vector containing E-tag peptide. Selection was performed in two consecutive rounds of selection. For the first round, 1 μg of C1q in 100 μL PBS was coated in one well of a Maxisorp 96-well plate at 4°C ON. Following, the well was added 300 μL PBS containing 2% bovine serum albumin (BSA) and incubated at RT for 1 h. Simultaneously, phages were blocked in 100 μL PBS containing 2% BSA for 1 h at RT. The well was washed with 3 × 300 μL PBS containing 0.1% Tween-20 (PBS-T) and phages were added and incubated for 1 h at RT. The well was washed with 15 × 300 μL PBS-T followed by 15 × 300 μL PBS and phages were eluted by addition of 100 μL 0.2 M glycine pH 2.2. Phages were neutralized by addition of 15 μL Tris pH 9.1 and used for infection of ER2738 cells. Cells were plated on 2xYT agar plates containing 100 μg/mL ampicillin and 2% glucose and incubated ON at 30°C. The following day the cells were recovered in 2xYT media and used for generation of phage particles with VCSM13 helper phages. The second round of selection was performed essentially as the first round of selection but now using 0.1 μg C1q. Following this round of selection, single colonies from 2xYT agar plates were inoculated into a 96-well plate containing 200 μL LB media with 100 μg/mL ampicillin. The plate was incubated at 37°C for 8 h, added IPTG to a final concentration of 1 mM and incubated ON at 30°C. The following day the plate was centrifuged for 10 min at 450 g and the supernatant was used in an ELISA as described (34). Phagemids from ELISA positive clones were isolated and sequenced.

### Expression and Purification of C1qNb75

The gene encoding C1qNb75 was cloned into a pET22b+ vector and transformed into LOBSTR cells (68). Cells were grown in LB medium containing 100 μg/mL ampicillin and 0.2% glucose at 37°C. At OD600 = 0.6–0.9 IPTG was added to a final concentration of 0.5 mM and incubated at 20°C ON. Cells were harvested by centrifugation and resolubilized in PBS containing 500 mM NaCl and 20 mM imidazole (wash buffer) pH 8.0. Cells were sonicated and centrifuged at 14,000 g for 30 min. The supernatant was incubated with Ni-NTA agarose beads (Protino), washed with wash buffer and eluted with wash buffer containing 300 mM imidazole pH 7.5. The protein was dialyzed against 50 mM sodium acetate pH 5.5 and loaded on a MonoS 5/10 column (GE Healthcare) equilibrated in 50 mM sodium acetate pH 5.5. The protein was eluted with a gradient from 0 to 500 mM NaCl and analyzed by SDS-PAGE. Fractions containing C1qNb75 were concentrated and loaded on a Superdex75 10/300 column (GE Healthcare) equilibrated in 20 mM HEPES-NaOH, 150 mM NaCl, pH 7.5. The final yield was approximately one mg/L LB media.

### Classical Pathway Deposition Assays

Each well in a 96-well Maxisorp plate (Thermo, Catalog Nr. 446612) was coated with 100 μL solution of heat aggregated IgG at 15 μg/mL in 50 mM sodium carbonate (pH 9.6) (AMPLIQON Laboratory Reagents) and incubated ON at 4°C. The wells were blocked by addition of 250 μL TBS/Tw (20 mM Tris, 150 mM NaCl pH 7.4 and 0.05 % Tween-20) supplemented with 1 mg/mL human serum albumin (HSA) for 1 h at RT. The wells were washed three times with 300 μL 20 mM HEPES-NaOH, 150 mM NaCl, pH 7.5. Nanobodies were diluted in veronal buffer (4.9 mM barbital, 145 mM NaCl, 0.25 mM CaCl_2_, 1.76 mM MgCl_2_ pH 7.28-7.6) (LONZA, Catalog No. 12-624E) placed on ice and added NHS to a final concentration of 0.2%. The diluted Nb/NHS (100 μL) mixture was transferred to IgG coated wells and incubated for 90 min at 37°C in a humidity chamber. The wells were subsequently washed three times with TBS/Tw (TBS with 0.1% (v/v) Tween 20). Hundred microliter biotinylated rabbit anti-C3c antibody (Dako, A0062) or biotinylated rabbit anti-C4c antibody (Dako, Q0364) diluted to 0.5 μg/mL in TBS/Tw was added and the plates were incubated for 1 h at RT. The wells were washed three times in TBS/Tw and each well was added 100 μL of 1 μg/mL europium-labeled streptavidin (PerkinElmer, Catalog No. 1244-360) diluted in TBS/Tw containing 25 μM EDTA and incubated for 1 h at RT. The wells were washed three times in TBS/Tw and then 200 μL enhancement buffer (AMPLIQON lab reagent, Catalog No. Q99800) was added to each well. Time-resolved fluorescence was subsequently measured using a VICTOR3 Multilabel Plate counter (PerkinElmer).

### Binding of C1q to Nanobodies

Wells of Maxisorp plates were coated with 100 μL solution of nanobody or with rabbit F(ab′)_2_ anti-human C1q (the F(ab′)_2_ was prepared by pepsin treatment of rabbit anti-C1q antibody (A136, Dako) at 10 μg/mL in PBS and incubated ON at 4 °C. The wells were blocked by addition of 250 μL TBS supplemented with 1 mg/mL HSA for 1 h at RT. The wells were washed three times with TBS/Tween. Serum or purified C1q were diluted in TBS/Tw/Ca (TBS/Tw containing 5 mM CaCl_2_) and 100 μL added per well. Following incubation for 1 h the wells were washed three times with TBS/Tw and 100 μL of biotinylated rabbit anti-C1q antibody (Dako A136, biotinylated by standard methods) diluted to 0.25 μg/mL in TBS/Tw and the plates were incubated for 1 h at RT. The wells were washed three times in TBS/Tw and each well the received 100 μL of 1 μg/mL europium-labeled streptavidin diluted in TBS/Tw containing 25 μM EDTA and incubated for 1 h at RT. The wells were washed three times in TBS/Tw and then 200 μL enhancement buffer was added to each well and time-resolved fluorescence was measured using a VICTOR3 Multilabel Plate counter (PerkinElmer).

### Binding of C1q to IgG in the Presence of C1qNb75

Wells of Maxisorp plates were coated with 100 μL of normal human IgG at 15 μg/mL in in 50 mM sodium carbonate (pH 9.6) and incubated ON at 4°C. The wells were then blocked by addition of 250 μL TBS supplemented with 1 mg/mL HSA for 1 h at RT. The wells were then washed three times with TBS/Tw. Purified C1q (final concentration 50 ng/mL), diluted in TBS/Tw/Ca, was mixed with nanobodies or with rabbit anti-C1q at several dilutions and 100 μL added per well. Following incubation for 1 h, the wells were washed three times with TBS/Tw and then 100 μL of biotinylated rabbit anti-C1q antibody diluted to 0.25 μg/mL in TBS/Tw were added, and the plates were incubated for 1 h at RT. The wells were washed three times in TBS/Tw and each well then received 100 μL of 1 μg/mL europium-labeled streptavidin diluted in TBS/Tw containing 25 μM EDTA and incubated for 1 h at RT. The wells were washed three times in TBS/Tw and then 200 μL enhancement buffer was added to each well and time-resolved fluorescence was measured using a VICTOR3 Multilabel Plate counter (PerkinElmer).

### Assay for C5a in Serum

Serum samples with nanobody at different concentrations were incubated at 37°C for 18 h to initiate autoactivation of complement fragments. The samples were subsequently diluted 1/15 and tested for C5a content. The concentration of complement fragment C5a was measured using an ELISA kit from Hycult Biotech (catalog number HK349) as described by the manufacturer. It uses incubation of samples in microtiter wells coated with a monoclonal antibody that recognizes a neo-epitope on C5a. This is followed by detection of bound C5a with a biotinylated antibody and subsequently addition of enzyme-labeled streptavidin and enzyme substrate.

### Bio-layer Interferometry (BLI) Affinity Measurement

BLI experiments were performed on an Octet Red96 (ForteBio) at 30°C, 1,000 rpm. C1qNb75 were immobilized on amine reactive second-generation (AR2G) biosensors at 20 μg/mL in 20 mM sodium acetate, 100 mM NaCl pH 5.0. The sensors were quenched with 1 M ethanol amine and equilibrated in kinetics buffer (PBS supplemented with 1 mg/mL BSA and 0.05% Tween 20). Association and dissociation were monitored with gC1q concentrations ranging from 0.3 to 10. 9 nM and C1q concentrations ranging from 0.7 to 5.4 nM in kinetics buffer. For gC1q, the data were globally fitted with a 1:1 binding model using the Octet Data Analysis software.

### Binding of C1q to IgG on Pig Red Blood Cells in the Presence of C1qNb75

Venous EDTA stabilized blood was sampled from pigs undergoing experimental surgery at the Institute of Clinical Medicine, Aarhus University, Denmark and fixed with glutaraldehyde as described (32). Antibody deficient serum (ADS) was excess material from clinical investigations of a person with antibody deficiency and the use was approved by The Danish Data Protection Agency (reference number 1-16-02-40-12/2007-58-0010) and the Ethics Committee in Central Denmark Region (reference number 1-10-72-127-12). Human IgG antibodies against terminal galactose-alpha-1,3-galactose were affinity isolated from a normal human IgG pool as described (32). Primary incubation of cells, human antibody deficient serum, and antibody was performed in RPMI with HSA at 1 g/L constituting total volumes of 20 μL. The tubes were placed in a 37°C water bath and incubated for 2 h. Nanobodies or EDTA were added as stated. Red blood cells were then washed in PBS by centrifugation (200 g, 10 min). Cells were resuspended in 20 μL PBS containing HSA at 1 mg/mL and secondary antibody (stock solutions diluted 300-fold). Secondary antibodies were fluorescein-isothiocyanate (FITC)-labeled polyclonal rabbit F(ab')2 anti-human IgG or biotinylated polyclonal rabbit anti-human C1q (both from Dako, Denmark). Secondary incubations were done in the dark at RT for 30 min. RBCs were washed again in PBS/HSA and resuspended in 20 μL Streptavidin APC-eFluor780 (eBioscience/ThermoFisher Scientific, Waltham, MA) in PBS/HSA (v/v: 1/300). The cells were mixed and incubated in the dark at RT for 30 min. Finally, the cells were washed and resuspended in 100 μL flow buffer (PBS, pH 7.4) and analyzed on a NovoCyte Quanteon (ACEA Biosciences, Inc., San Diego, CA). Median fluorescence intensity (MFI) of experiments were subtracted background fluorescence and expressed relative to the mean of a reference experiment as stated in the text. Background fluorescence was defined as 99.9% of the lowest MFI acquired in associated experiments (99.9% was preferred over 100% to allow use of geometrical means).

### Hemolysis Assay With IgG Coated Sheep Red Blood Cells

Sheep red blood cells (sRBCs) in Alsever's solution (SSI, Denmark) were washed with BI buffer (Lonza veronal buffer containing 2 mM CaCl_2_) supplemented with 1 mg/mL gelatin and diluted to 6% v/v. Lyophilized rabbit anti-sRBC stroma antibody (Sigma-Aldrich S1389) was reconstituted in 2 mL PBS and 50 μL was added to 10 mL BI buffer. The antibody was mixed with 10 mL 6% sRBCs in BI buffer and incubated at RT for 30 min. Cells were pelleted by centrifugation at 1,000 g for 6 min and the supernatant was removed. The cells were resuspended in 10 mL BI buffer. For each Nb concentration, 15 μL of NHS was added to 185 μL Nb diluted in BI buffer and incubated for 15 min on ice. Sixty microliter from each reaction were then transferred to a V-shaped 96-well plate in triplicates and placed on wet ice before 30 μL 6% sRBCs were added. The plate was vortexed and incubated at 37°C in a humid chamber at 250 rpm. After 30 min, 100 μL stop solution (0.9% NaCl, 5 mM EDTA) was added to each well. The plate was vortexed and subsequently centrifuged at 450 g for 6 min. Fifty microliter of sample was transferred to a flat-bottom microtiter plate containing 50 μL stop solution and vortexed. The absorbance was measured at 405 nm using a VICTOR3 Multilabel Plate counter. % lysis was calculated with the formula: % lysis = ((OD405_test_-OD405_blank_)/(OD405_totallysis_-OD405_blank_))^*^100, with OD405_blank_ being incubation with buffer and OD405_totallysis_ incubation with water.

### Hemolysis Assay With IgM Coated Sheep Red Blood Cells

sRBCs coated with rabbit anti-sheep erythrocyte IgM (Complement technology, catalog #B200) were pelleted by centrifugation at 1,000 g. Supernatant was removed and sRBCs were resuspended in veronal buffer (Lonza) containing 4.9 mM barbital, 145 mM NaCl, 0.25 mM CaCl_2_, 1.76 mM MgCl_2_ pH 7.28–7.6 and supplemented with 0.1% gelatin (assay buffer) to 5 × 10^8^ cells/mL. For each reaction 20 μL Nb was mixed with 53 μL assay buffer and 100 μL NHS diluted 1/50 in assay buffer. After 15 min incubation on ice, 27 μL sRBCs was added and 90 μL from each reaction was pipetted into a V-shaped 96-well plate in duplicates. The plate was incubated in a water bath for 60 min at 37°C with shaking at 1,400 rpm every 10 min. After 60 min, the plate was centrifuged at 1,000 g for 3 min and 80 μL form each well was pipetted into a flat bottom 96-well plate and absorbance was measured at 541 nm. % lysis was calculated by using the following formula: % lysis =((OD541_test_-OD541_blank_)/(OD541_totallysis_-OD541_blank_))^*^100, with OD541_blank_ being sRBCs incubated with buffer and OD541_totallysis_ being sRBCs incubated with water. IC50 was calculated with GraphPad by fitting the data with a variable slope model.

### Generation of Stable Cell Line and Purification of Human gC1q

A transfected HEK293 stable cell line for expression of the single-chain form of gC1q (69) with a C-terminal His6 tag was generated as follows. Prior to transfection, HEK 293F cells were split to 0.5 × 10^6^ cells/mL in serum-free FreeStyle 293 medium (Gibco) and incubated ON at 37°C at 8% CO_2_ with shaking. The following day, cells were collected by centrifugation and resuspended in fresh medium to give 2 × 10^6^ cells/mL. The cells were incubated for 1 h with shaking and transfected using 1 μg DNA (gC1q in pcDNA3.1) per 1 mL final culture volume and a 2:1(*w*:*w*) ratio of polyethylenimine (25 kDa; Polysciences) to DNA. After 4 h, the cells were diluted to 10^6^ cells/mL in FreeStyle 293 medium and incubated ON at 37°C at 8% CO_2_ with shaking. The next day, 0.25 × 10^6^ viable cells from each of the transfected cell cultures were transferred to 6-well plates containing FreeStyle 293 medium supplemented with 10% (*v*/*v*) fetal bovine serum (FBS) and incubated statically ON at 37°C at 8% CO_2_ to allow the cells to adhere. Selection pressure was applied by the addition of fresh medium containing 10% FBS and 200–300 μg/mL Geneticin (Gibco). The medium was changed every 2–3 days to remove dying cells and selection pressure was maintained for 11–18 days until foci could be identified. Single colonies were picked to a 24-well plate and left to proliferate in FreeStyle 293 medium supplemented with FBS and Geneticin, before being transferred to 6-well plates and adapted to 0.1% FBS. Finally, the cells were resuspended in 10 mL FreeStyle 293 medium supplemented with 250 μg/mL Geneticin and transferred into 125 mL flasks. The cells were cultured as recommended by the manufacturer until a cell viability of >90% was achieved. A western blot was performed on 20 μL media to identify clones with high gC1q expression. The western blot was performed using THE^TM^ His-Tag Antibody, (Genscript) (diluted 1:3,000 in low fat milk) and Goat anti-mouse IgG HRP (Thermo Fisher Scientific) (diluted 1:15,000 in PBS-T). Four clones with high gC1q expression were selected and used for small scale expression and pulldown experiment using Ni Excel beads (GE healthcare). The best clone yielded ~2–4 mg protein/L culture.

For large-scale expression, gC1q expressing cells were grown in FreeStyle 293 media for 7 days at 37°C, 150 rpm and 8 % CO_2_. Cells were harvested by centrifugation and gC1q was purified from culture media adjusted to pH 8.0 using a 5 mL Histrap excel column (GE Healthcare). The column was washed to baseline with PBS containing 500 mM NaCl and 10 mM Imidazole pH 8.0. The protein was eluted with PBS containing 500 mM NaCl and 400 mM Imidazole pH 8.0 and analyzed by SDS-PAGE. Fractions containing gC1q were concentered and loaded on a Superdex 200 Increase 10/300 column (GE Healthcare) in 20 mM HEPES-NaOH, 150 mM NaCl, pH 7.5.

### Crystallization of hC1qGH-C1qNb75 Complex

For complex formation gC1q was mixed with molar excess of C1qNb75 and purified by size exclusion chromatography in 20 mM HEPES-NaOH, 150 mM NaCl, pH 7.5 using a BioRad ENrich SEC 70 10/300 column. Complex containing fractions were pooled and concentrated to 8 mg/mL. The complex was crystallized at 19°C by vapor diffusion against a reservoir containing 0.2 M ammonium acetate, 0.1 M BIS-Tris pH 5.5, 25% w/v PEG 3350 after mixing protein and reservoir in a 1:1 ratio. Crystals were cryoprotected in 0.1 M BIS-Tris pH 5.5, 37% w/v PEG 3350 and cryocooled in liquid nitrogen.

### Data Collection and Structure Determination

Diffraction data were collected to 2.19 Å at the EMBL P14 beam line, PETRA III. Diffraction data from a single crystal were processed with XDS (70). The structure was determined by molecular replacement using Phaser (71) taking advantage of the single chain gC1q structure (PDB ID: 5HKJ) and a nanobody (PDB ID: 5NLW) as search models. In an iterative manner, the structure was rebuilt in COOT (72) and refined using phenix.refine (73).

### C1-IgM-C4b Model Building

The C1-IgM model was built in the deposited cryo-ET 3D reconstruction (EMDB-4878) (17) using Pymol version 2.3.0. The Fc hexamer was derived from the crystal packing of PDB entry 1HZH (74). Next, the 6FCZ entry containing the gC1q and the IgG-Fc was superimposed on each Fc in the 1HZH hexamer. For construction of the hexameric IgM-C1 model, the 6FCZ hexamer was manually docked into EMDB-4878 density. The gC1q's were translated outwards to fit the density without rotation, implying that the gC1q's arrangement is wider in the IgM bound C1 complex (17). To model the two paired Cμ3 domains, the corresponding Cε3 dimer from the structure of IgE Fc [PDB entry 2WQR, (75)] was docked into the density. The model was completed by placing at each docked IgE Cε3 domain an omalizumab Fab from PDB entry 2XA8 (76). The structure of MASP-2 CCP1-CCP2-SP domains in complex with C4b (PDB: 4FXG) was placed inside the density and the C345c domain was separated from the rest of C4b in order to improve the fitting while maintaining the contacts with MASP-2. Next, the MASP-2 domains were replaced by C1s (PDB: 5UBM) (77) and C1r CCP1-CCP2-SP domains (PDB: 2QY0) were fitted in the corresponding density (78). The tetramer formed by the C1r and C1s CUB1-EGF-CUB2 domains (PDB: 6F1C) (79) was fitted in the core by separating the two heterodimers and applying a translation, while the C1s CUB2 domain was released from the structure and fitted separately into the density. The C1q collagen stems generated as in (35) but with one additional interruption in the collagen fold at residues A88, B93, C92, were subsequently added in the density. A list of the PDB files used is provided in Table S2.

## Data Availability Statement

Diffraction data and the atomic model are deposited at the protein data bank PDBe under the entry 6Z6V.

## Author Contributions

NL, DP, HG, AZ, JB, and AH generated C1qNb75, expressed and purified proteins, and determined the structure or conducted functional assays. ST and GA supervised research. NL and GA wrote the manuscript with inputs from all authors. All authors contributed to the article and approved the submitted version.

## Conflict of Interest

NL, AZ, DP, ST, and GA are listed as inventors on a patent describing the use of C1qNb75. The remaining authors declare that the research was conducted in the absence of any commercial or financial relationships that could be construed as a potential conflict of interest.
