# Supplementary material for: Functional and Structural Characterization of a Potent C1q Inhibitor Targeting the Classical Pathway of the Complement System
Source: Front Immunol. 2020 Jul 17;11:1504. doi: 10.3389/fimmu.2020.01504 (PMC7396675; doi:10.3389/fimmu.2020.01504)
Supplement: Supplementary file 1 [file Data_Sheet_1.docx]

Supplementary Material

**Figure S1. C4 fragment deposition in the presence of nanobodies, evaluation of fluid phase activation of complement by C1qNb75 and comparison of C1qNb75 bound gC1q with unbound gC1q.** (**A**) Deposition of C4 fragments on IgG coated surface from NHS in the presence of the indicted concentrations of C1q nanobodies and control nanobody. Shown are mean of two replicates and SD. (**B**) Fluid phase activation of complement by C1qNb75 measured by C5a generation demonstrates that the nanobody does not trigger fluid phase activation. (**C**) Alignment of gC1q (colored as in Fig. 4) in complex with C1qNb75 with the structure of single chain gC1q (grey, PDBID 5HKJ).

**Figure S2. Sequence alignment of the C1qNb75 epitope in human C1q B with mouse, pig and macaque gC1q B, homology model of murine gC1q, SDS-PAGE of purified hC1q and recombinant gC1q, and sequence of C1qNb75.** (**A**) Sequence alignment of the C1q B epitope region from man and mouse, pig and macaque. C1q B residues forming direct contacts in the structure are labeled by triangles, contact residues differing between the four species are boxed in red. The degree of blue shading denotes conservation. (**B**) To the left is shown a view or the hgC1q:C1qNb75 complex of a contact area with little conservation between human and mouse C1q B. To the right is presented a close-up view of the hydrophobic pocket housing the W105 in a hypothetical model of a complex between mouse gC1q and C1qNb75. The lysine present in mouse gC1q B is too large to fit in the interface and bears a full positive charge. The mouse gC1q A, B and C chains were built by homology using SWISS-MODEL and aligned to the hgC1q corresponding chains. The chains are colored as in Figure 4. (**C**) SDS-PAGE of purified C1q and recombinant single chain gC1q. The bands for the A, B and C-chains of C1q are labeled. (**D**) Protein sequence of C1qNb75 including the C-terminal His-tag.

**Table S1. X-ray data collection and refinement statistics for gC1q in complex with C1qNb75.**

| **Data Collection** | |
| --- | --- |
| Resolution range | 57.72 - 2.188 (2.267 - 2.188) |
| Space group | P 2 21 21 |
| Unit cell | 61.816 119.744 131.772 90 90 90 |
| Total reflections | 618207 (15326) |
| Unique reflections | 48574 (2516) |
| Multiplicity | 12.7 (6.1) |
| Completeness (%) | 94.69 (50.05) |
| Mean I/sigma(I) | 15.70 (1.98) |
| Wilson B-factor | 38.53 |
| R-merge | 0.1172 (0.7639) |
| R-meas | 0.1221 (0.8346) |
| R-pim | 0.03371 (0.3159) |
| CC1/2 | 0.999 (0.628) |
| **Refinement** | |
| Reflections used in refinement | 48560 (2516) |
| Reflections used for R-free | 2000 (104) |
| R-work/R-free | 0.1777 (0.2654)/0.2267 (0.3445) |
| Number of non-hydrogen atoms | 8476 |
| macromolecules | 8116 |
| ligands | 28 |
| Protein residues | 1038 |
| RMS (bonds) | 0.01 |
| RMS (angles) | 1.39 |
| Ramachandran favored, allowed, outliers (%) | 95.98, 3.92, 0.1 |
| Clash score | 2.19 |
| Average B-factor | 46.78 |

**Table S2. Table of PDB files used for generating the model of C1 in complex with IgM and C4b.**

| **PDB ID** | **Description** |
| --- | --- |
| 2XA8 | Fab-Cµ2 fragments |
| 2WQR | IgM Cµ3 paired domains |
| 1HZH, 6FCZ | IgM-Fc hexamer |
| 6FCZ | gC1q |
