# Supplementary figures and images for: Functional and Structural Characterization of a Potent C1q Inhibitor Targeting the Classical Pathway of the Complement System
Source: Front Immunol. 2020 Jul 17;11:1504. doi: 10.3389/fimmu.2020.01504 (PMC7396675; doi:10.3389/fimmu.2020.01504)

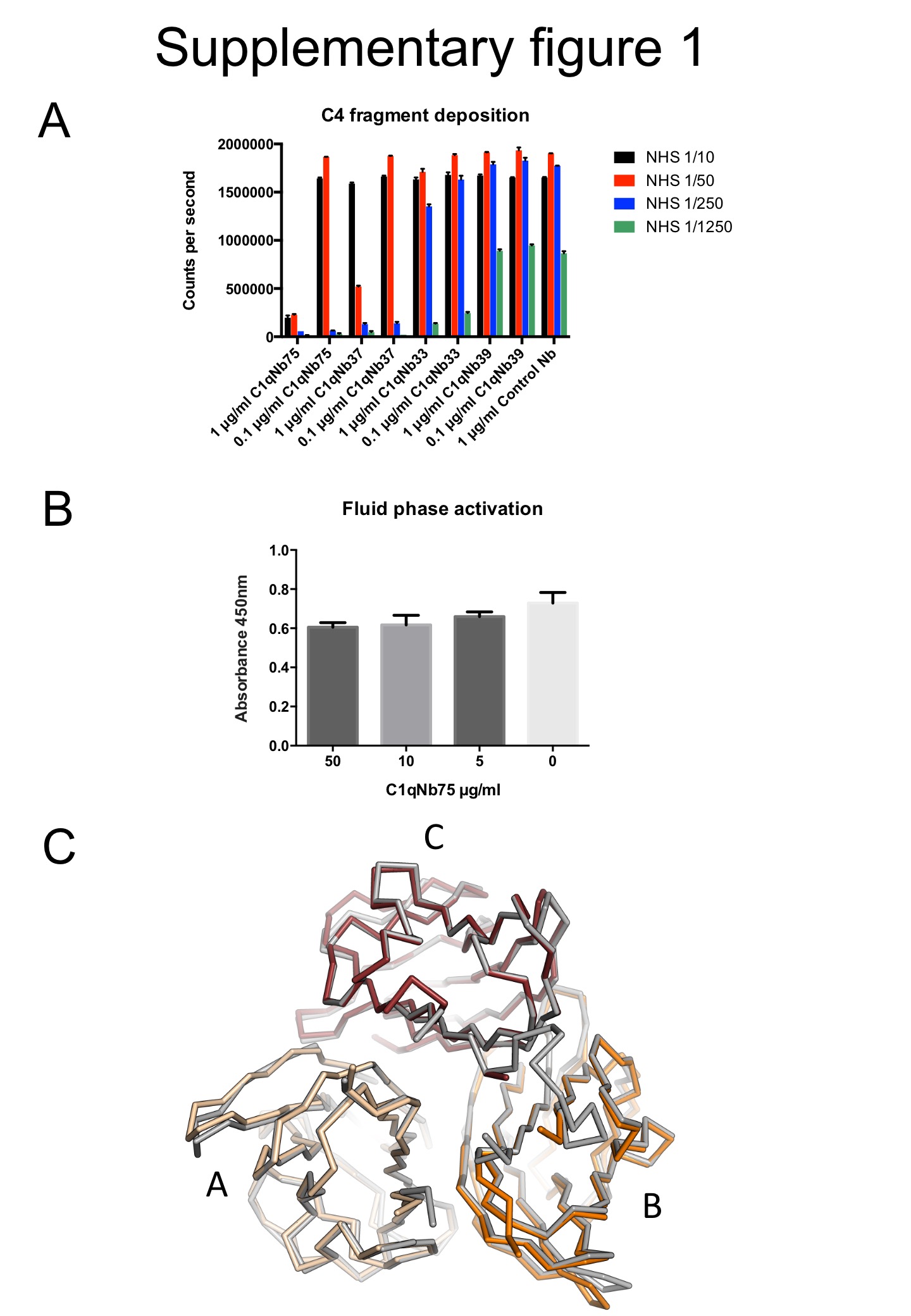

Supplement: Supplementary file 2 [file Image_1.jpeg]

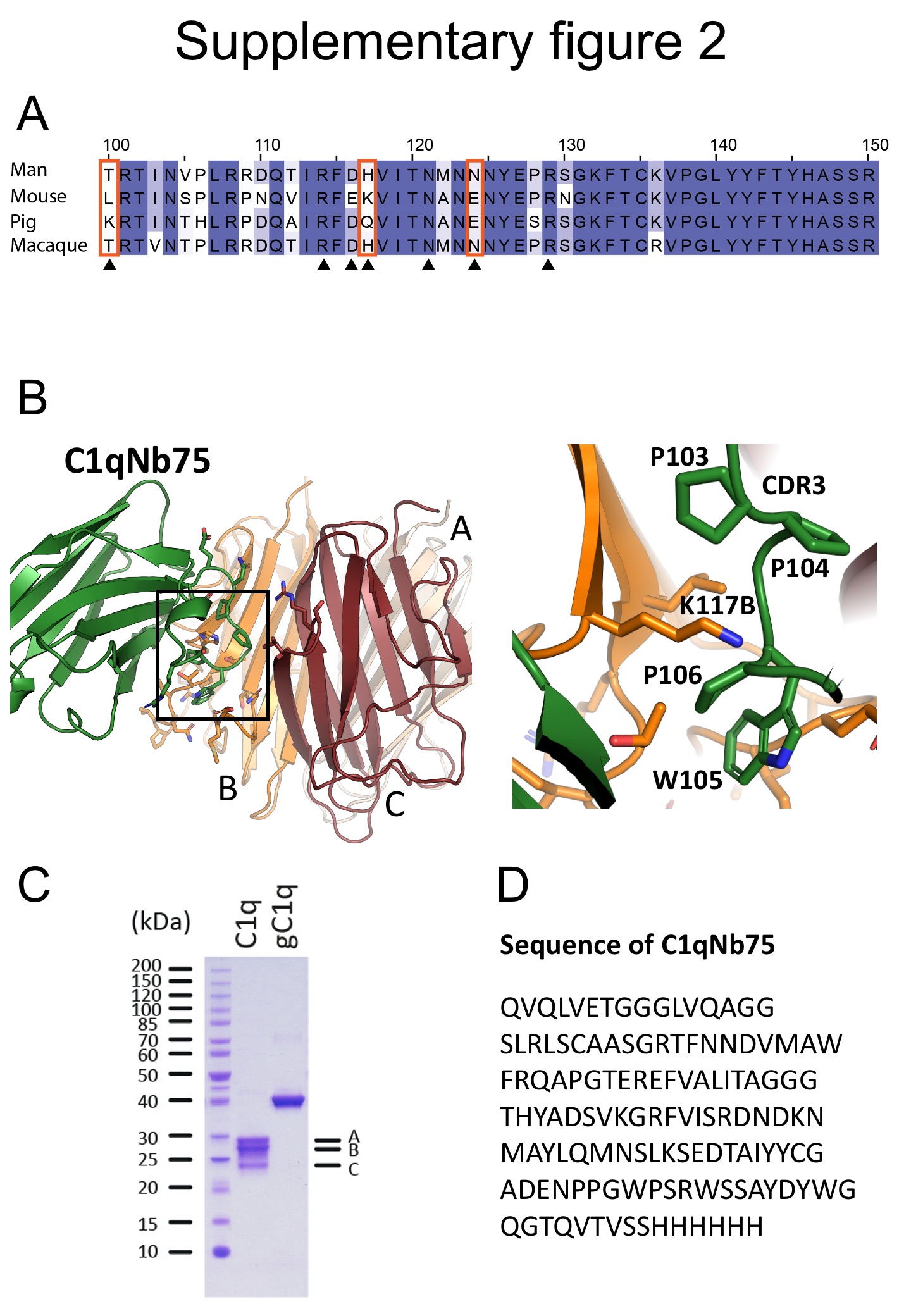

Supplement: Supplementary file 3 [file Image_2.jpeg]
